# Evaluation of the potential role of long non-coding RNA LINC00961 in luminal breast cancer: a case–control and systems biology study

**DOI:** 10.1186/s12935-020-01569-1

**Published:** 2020-10-02

**Authors:** Sepideh Mehrpour Layeghi, Maedeh Arabpour, Rezvan Esmaeili, Mohammad Mehdi Naghizadeh, Javad Tavakkoly Bazzaz, Abbas Shakoori

**Affiliations:** 1 Department of Medical Genetics, School of Medicine, Tehran University of Medical Sciences, Tehran, Iran; 2 Genetics Department, Breast Cancer Research Center, Motamed Cancer Institute, ACECR, Tehran, Iran; 3 Noncommunicable Diseases Research Center, Fasa University of Medical Sciences, Fasa, Iran; 4 Medical Genetic Ward, Imam Khomeini Hospital Complex, Tehran University of Medical Sciences, Tehran, Iran; 5 Breast Disease Research Center (BDRC), Tehran University of Medical Sciences, Tehran, Iran

**Keywords:** Breast cancer, LINC00961, Luminal A, Luminal B, Bioinformatics analysis

## Abstract

**Background:**

Luminal subtype is the most common subgroup of breast cancer (BC), accounting for more than 70% of this cancer. Long non-coding RNAs (lncRNAs) are a group of RNAs which play critical roles in diverse cellular processes. It is proved that dysregulation of them can contribute to the development of various cancers, including BC. LINC00961 was reported to be downregulated in several cancers, however, its expression level in BC remains largely unknown. The purpose of the present study was to investigate the possible role of LINC00961 in luminal A and B subtypes of BC.

**Methods:**

To obtain novel lncRNAs associated with different cancers and differentially expressed lncRNAs (DElncRNAs) between BC tumor and normal tissues, Lnc2Cancer and GDC databases were used, respectively. After performing literature review, the expression level of the selected lncRNA (LINC00961) was evaluated in 79 luminal A and B BC specimens and adjacent non-cancerous tissues by Quantitative Reverse Transcription PCR (qRT-PCR). LINC00961 expression was also evaluated in two luminal A BC cell lines, compared to a normal breast cell line. The comparison of the differences between tumor and adjacent non-tumor samples was performed by paired sample t-test. Moreover, correlation analysis between LINC00961 expression and clinicopathological features was performed using the chi-square, fisher exact, and independent t-test. In order to investigate the possible roles of LINC00961 in luminal A and B BC, different bioinformatics analyses such as functional annotation of the LINC00961 co-expressed genes and protein–protein interaction (PPI) networks construction were also performed.

**Results:**

LINC00961 was selected as a significant DElncRNA which had not been studied in BC. According to q-RT PCR assay, LINC00961 was downregulated in luminal BC tissues and cell lines. Its expression was correlated with smoking status and the age of menarche in luminal BC patients. Also, the results of the bioinformatics analysis were consistent with the data obtained from q-RT PCR assay. The final results indicated that LINC00961 might be involved in multiple cancer-associated pathways such as chemokine, Ras and PI3K–Akt signaling pathways, GPCR ligand binding, and signal transduction in luminal subtypes of BC. CDH5, GNG11, GNG8, SELL, S1PR1, CCL19, FYN, ACAN, CD3E, ACVRL1, CAV1, and PPARGC1A were identified as the top hub genes of the PPI networks across luminal subgroup.

**Conclusion:**

Our findings suggested that LINC00961 was significantly downregulated in luminal A and B subtypes of BC. Moreover, bioinformatics analysis provided a basis for better identification of the potential role of LINC00961 in luminal subtype of BC.

## Background

As breast cancer (BC) is one of the most common human neoplasms, it poses major financial challenges to health systems and families of patients globally [1, 2]. It is also the second cause of cancer death among women worldwide [3]. Considering the status of estrogen (ER), progesterone (PR), HER2 receptors, and ki-67 levels, different intrinsic subtypes of BC include luminal A, luminal B, triple negative (TNBC), HER2/neu positive, and normal-like breast cancer [4]. Luminal A subtype is ER^+^/PR^+^/HER2^−^ and presents low levels of ki-67. The luminal A subtype has the highest frequency (50%) of invasive breast cancer, showing the best prognosis among all of BC subtypes. However, luminal B with positive expression of ER/PR and variable expression of HER2/neu constitutes 20% of invasive breast cancers. This subgroup showed worse prognosis and higher levels of ki-67 than the luminal A subtype [4]. It has been showed that survival and recurrence rates were different among luminal A and B subtypes of BC. Luminal A is associated with longer overall survival compared to luminal B. The recurrence rate was at 6.8%, and 5.3% in luminal B and luminal A, respectively [5]. The fact that luminal A and B subgroups together account for the highest frequency of invasive breast cancer as well as the limited number of studies conducted specifically on these two subgroups, increases the importance of further studies on these groups.

Long non-coding RNAs (lncRNAs) include a class of non-coding RNAs with a length of more than 200 nucleotides [6–8]. The lncRNAs are involved in a variety of processes, including chromatin rearrangement, gene regulation, and alternative splicing of multiple genes [9]. Their effect on the process of gene expression can occur at different levels, such as transcription, post-transcription, and translation stages [10]. In most cases, their altered expression can lead to the development of different human malignancies, including cancers. LncRNAs are key players in survival, proliferation, invasion, metastasis and resistance to therapy of cancer cells [11–13]. They can act as tumor suppressors or oncogenes [7, 14], and have a direct or indirect effect on tumorigenesis through regulation of cancer signaling pathways [12]. Also, some lncRNAs have been shown to have small open reading frames (ORFs) which enable them to generate functional polypeptides [15]. Abnormal expression of lncRNAs in cancer cells, high tissue specificity, and easy detection of them in tissues and body fluids make them an ideal diagnostic and prognostic marker in various cancers. Some lncRNAs have also been used to increase the specificity and sensitivity of other biomarkers [12].

LINC00961 (SPAAR), located in chromosome 9 (9p13.3), was found to act as a tumor suppressor in various cancers, such as oral squamous cell carcinoma (OSCC) [16], skin melanoma [17], and colon cancer [18]. In hepatocellular carcinoma (HCC), low expression of LINC00961 was significantly correlated with larger tumor size, lymphatic metastasis, and advanced stages of tumors [19]. Also, decreased expression of LINC00961 has been shown to be associated with shorter overall survival of glioma and non-small cell lung carcinoma (NSCLC) patients [20, 21]. According to the role of LINC00961 in different cancers that has been proved in previous studies, LINC00961 could play crucial roles in tumor suppression in various cancers. It has recently been determined that LINC00961 encodes a conserved polypeptide, named small regulatory polypeptide of amino acid response (SPAAR) which modulates skeletal muscle regeneration [15]. Although, the expression pattern and biological function of LINC00961 have been studied in many human cancers, to the best of our knowledge, the present study is the first project that investigates the role of this lncRNA in luminal BC so far.

In this research, the expression level of lncRNA LINC00961 was examined in luminal A and B BC tissues and luminal A cell lines compared with non-tumoral ones by qRT-PCR. Then, the correlation between expression level of LINC00961 and the clinicopathological features were explored. Moreover, bioinformatics and systems biology studies were accomplished.

## Materials and methods

### TCGA gene profiling data analysis and literature review

Lnc2Cancer database was used in order to identify novel lncRNAs associated with various cancers. This database provides 4989 lncRNA-cancer correlations between 1614 lncRNAs and 165 cancer subgroups [22]. Also, a project from the Cancer Genome Atlas (TCGA) BC RNA sequencing dataset was downloaded from the Genomic Data Commons (GDC) database [23]. The data of luminal A and B subtypes of BC was selected from the entire BC RNA sequencing dataset. Then, it was used for the discovery of DElncRNAs in luminal subgroup of BC. The comparison between tumoral and normal tissues makes it possible to identify the differentially expressed genes in various RNAseq datasets. Data analysis and p-value adjustment (padj.) were performed using DESeq2 R package. As a result, according to the obtained lncRNAs from both Lnc2Cancer and GDC database, a list of DElncRNAs was selected with |log2FC| > 1 and padj. < 0.05. Finally, a comprehensive PubMed search of the selected DElncRNAs was performed to determine the lncRNAs that had not been studied in BC.

### Patient samples

In the current case–control study, 79 paired tissues (tumoral and adjacent non-tumoral) were obtained from luminal BC patients who had not received any chemotherapy or hormone therapy prior to surgery. The samples were collected from Breast Cancer Research Center Biobank (BCRC-BB) [24]. BCRC-BB is required to follow ethical guidelines and suggestions for biobanks in storing and using human biological samples. This study was approved by the Ethics committee of Tehran University of Medical Sciences (TUMS) (Code of Ethics: IR.TUMS.MEDICINE.REC.1398.792). In addition, prior to entering the biobank, written consent was obtained from all patients. Also, the collected tumoral specimens have been pathologically approved by histopathological analysis. Clinical features including age at diagnosis, tumor size, subtype, grade, stage, pathology of tumors, ER/PR/HER2 status, smoking, ki-67 level, p53, body mass index (BMI), and age of menarche were attained from hospital records.

### Cell culture

Two luminal A (ER/PR^+^, HER2^−^) BC cell lines named MCF-7 and T47D were cultured in DMEM (Sigma-Aldrich, St. Louis, MO, USA) added with 10% fetal bovine serum (Gibco, Carlsbad, CA, USA), 100 U/ml penicillin, and 100 μg/ml streptomycin (Sigma-Aldrich, St. Louis, MO, USA). MCF-7 is a human epithelial breast adenocarcinoma cell line, while T47D is a human epithelial breast ductal carcinoma cell line. MCF10-A, a normal breast cell line, was maintained in DMEM supplemented with 5% fetal bovine serum, epidermal growth factor (EGF) (20 ng/ml), insulin (10 µg/ml), hydrocortisone (0.5 mg/ml), cholera toxin (100 ng/ml), penicillin (100 U/ml), and streptomycin (100 μg/ml). The cells were incubated in an incubator containing 5% CO_2_ at 37 °C.

### RNA extraction and qRT-PCR assay

The total RNA of luminal A and B breast tissues (tumoral and non-tumoral) and luminal A cell lines were extracted using RiboEx™ (GeneAll), following the manufacturer's instructions. Then, the obtained RNA was dissolved in diethyl pyrocarbonate-treated (DEPC) water. Next, RNA quality and quantity were checked using gel electrophoresis and spectrophotometry, respectively. The samples were treated with RNase inhibitor and afterwards, 1 µg of RNA samples was converted into cDNA using the Biobasic cDNA Synthesis Kit (5× All-In-One RT MasterMix) in accordance with the manufacturer’s protocol. Finally, qRT-PCR analysis was performed in duplicate on LightCycler96 Roche by utilizing AMPLIQON Real Q Plus 2× Master Mix Green low ROX. The condition of qRT-PCR was set at 95 °C for 15 min, and 40 cycles of 95 °C for 10 s and 60 °C for 25 s. The annealing temperature was determined by obtaining the temperature at which each pair of primers had the best efficiency. β2M was used as a reference gene. Data was analyzed and calculated using the 2^−ΔΔCt^ method. All used primer sequences were listed in Table 1.

**Table 1:** Primers for real‐time PCR

Genes	Forward (5′-3′)	Reverse (5′-3′)
β2M	AGATGAGTATGCCTGCCGTG	GCGGCATCTTCAAACCTCCA
LINC00961 (SPAAR)	GGGAATCTGTGCTCACGTCTT	TGGCCCCACTGCTGAAATC

*β2M* Beta-2-microglobulin, *PCR* polymerase chain reaction

### Bioinformatics analysis

#### Analysis of LINC00961 expression levels across normal breast tissue samples

The discovery of lncRNA expression patterns provides fundamental information for realizing the biological functions of lncRNAs in cells. To this end, the differential expression analysis of lncRNA LINC00961 was explored using different databases.

First, the Expression Atlas was used to check the expression of LINC00961 in normal breast tissues. The Expression Atlas is an open science resource that provides a comprehensive representation of gene expression pattern in various tissues or cell types of different species by using microarray and RNA‐seq datasets. TPM (transcripts per million) and FPKM (fragments per kilobase of exon model per million reads mapped) are used for estimating gene expression in this software [25]. Then, GENEVESTIGATOR software was used for estimating the expression of LINC00961 in normal breast tissues. GENEVESTIGATOR is a high-performance tool that integrates thousands of microarray and RNA-Seq experiments in order to exhibit gene expression across different biological conditions, such as cancers. Expression thresholds are calculated using all expression values of genes over studied samples for the platform in use. "LOW", "MEDIUM", and "HIGH" refer to the first quartile, interquartile and fourth quartile ranges, respectively [26].

#### Analysis of LINC00961 expression levels across breast cancer samples and cell lines

GEPIA (Gene Expression Profiling Interactive Analysis), an application based on the Genotype-Tissue Expression (GTEx) and TCGA datasets, was utilized for obtaining gene expression differential analysis in luminal A and B BC [27]. Then, LINC00961 expression was evaluated across 12 cancer categories, including 1910 luminal samples, using GENEVESTIGATOR software. Also, its expression was assessed in 13 and 3 luminal BC and non-malignant breast cell lines, respectively [26].

#### Correlation analysis of co-expressed genes with LINC00961

FPKM file of 420 luminal BC samples, including 230 luminal A and 190 luminal B samples, were downloaded from the GDC database and a list of co-expressed genes with LINC00961 across the mentioned samples was obtained. Pearson correlation analysis of the obtained LINC00961 co-expressed genes has been computed using a standard method. Then, analyzing the expression of LINC00961 and its co-expressed genes across luminal A and B Breast invasive carcinoma (BRCA) dataset was done by the GEPIA web server [27].

#### Evaluation of the genetic alterations underlying breast cancer

The Cancer Genome Atlas (TCGA) copy number portal was used to search for the somatic copy number alterations (SCNA) of LINC00961. The TCGA is a broad portal that helps increasing our knowledge of the molecular basis of different cancers by investigating the SCNA of different genes which drive cancer progression. It includes information on 33 different cancers from 11,328 patients [28]. Also, genomic alterations of LINC00961 were evaluated by the International Cancer Genome Consortium (ICGC). The ICGC is a comprehensive portal for the detection of genomic abnormalities across 50 different cancer types and facilitates visualizing and downloading different cancer data sets [29].

#### Analysis of LINC00961 expression in different BC subtypes

Expression of LINC00961 in different subtypes of BC was evaluated by TANRIC database [30].

#### Functional annotation analysis

DAVID (The Database for Annotation, Visualization and Integrated Discovery) is an integrated bioinformatics resource which assists in deep understanding of biological mechanisms by providing an expansive set of functional annotation tools [31, 32]. Gene Ontology (GO) term enrichment analysis was performed using the obtained list of LINC00961 co-expressed genes across luminal A and B samples of BC in the previous step. At this level, REVIGO was used to facilitate the interpretation of the obtained Gene Ontology terms. REVIGO performs this by reducing redundancy of GO terms and prioritizing them based on the statistical significance of the terms [33]. Then, Cytoscape software was used for visualizing networks of files previously obtained from REViGO [34]. Finally, the STRING database was used to determine and visualize PPIs of the co-expressed genes with LINC00961 [35]. In addition, the Degree method provided in CytoHubba, a Cytoscape application, was used to explore the hub genes in the PPI network of the LINC00961 co-expressed genes [36].

### Statistical analysis

The obtained data were analyzed using IBM SPSS Statistics (Statistical Package for the Social Sciences, Version 24.0). Paired sample t-test was used for comparison of the differences between the means of tumoral and adjacent non-tumoral samples. The data is represented as mean ± standard deviation (SD). In order to perform clinicopathological correlation analysis, median expression of LINC00961 was used as a cut-off value for dividing 79 BC patients into two categories: relatively high expression of LINC00961 (n = 39) and relatively low expression of LINC00961 (n = 40). The chi-square and Fisher exact test for qualitative and independent t-test for quantitative variables were used to determine the difference of clinicopathological characteristics between low and high expression groups of LINC00961. Moreover, the receiver operating characteristic (ROC) curve was illustrated by IBM SPSS Statistics. This curve was used to evaluate the diagnostic effectiveness and clinical value of LINC00961 in luminal A and B subtypes of BC. p-value < 0.05 was considered as significant.

## Results

### LINC00961 was selected as a novel DElncRNA in breast cancer

According to the lnc2Cancer database, a list containing more than 4000 important lncRNAs associated with different cancers was obtained. Also, lists of 1178 and 1266 different DElncRNAs in luminal A and B BC paired tissues (tumoral and normal specimens) were obtained, respectively. According to these two lists of lncRNAs and literature review, LINC00961 was selected as a target lncRNA of the present study (logFC_luminal A_ = − 2.08, padj. = 8.45E−14) (logFC_luminal B_ = − 2.87, padj. = 1.99E−18).

### LINC00961 was significantly downregulated in luminal A and B BC tissues and cell lines

**Fig. 1:**
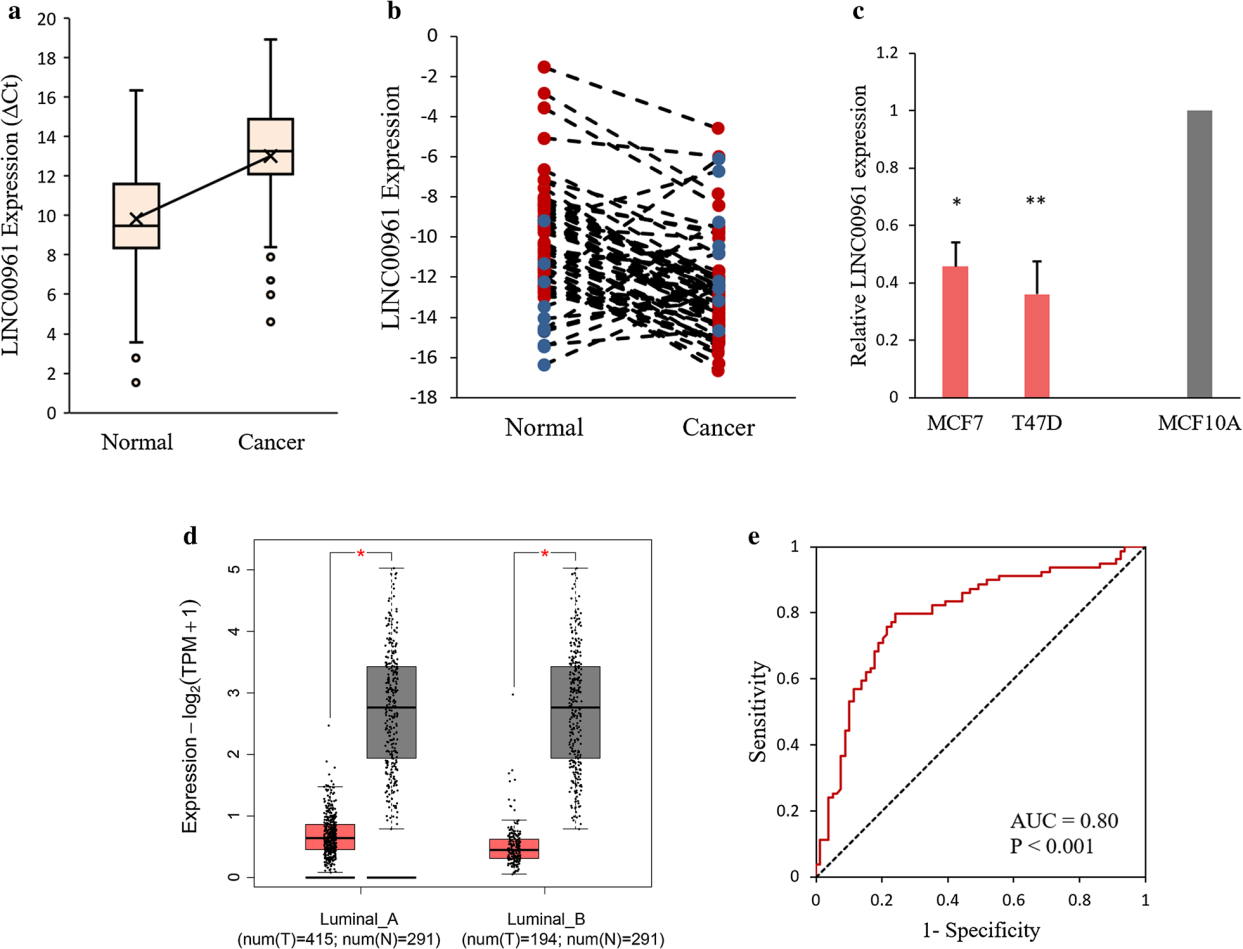
LINC00961 was downregulated in luminal A and B BC. **a **The expression level of LINC00961 detected by qRT-PCR in 79 luminal BC tissues and matched adjacent non-tumoral tissues. Results were presented as ΔCt in cancer tissues relative to adjacent tissues. **b** The relative expression of LINC00961 in two BC cell lines compared with normal breast cell line. **c** Downregulation of LINC00961 in 86% of samples compared to their adjacent non-tumoral tissues. Red bullets are representative of samples which LINC00961 was downregulated in. B2M was used as an internal control. Data were presented as the mean ± SD. *p-value = 0.008, **p-value = 0.010. *β2M* Beta-2-microglobulin. **d** Expression analysis of LINC00961 across BRCA in luminal A and B tumoral and normal samples, retrieved from the GEPIA web server. Red and gray colors are representative of tumor and normal samples, respectively. The expression levels were calculated using the log2(TPM + 1) scale. **e** ROC curve of LINC00961 in luminal BC. The X axis, “1-Specificity”, represents false positive rate and the Y axis, “Sensitivity”, displays true positive rate

According to the qRT-PCR results, LINC00961 was significantly downregulated in luminal A and B BC tissues compared with adjacent non-tumoral tissues (p-value < 0.001, Fig. 1a). The mean expression level of LINC00961 was not notably different between luminal A and luminal B subtypes of BC patients (p-value > 0.05). As shown in Fig. 1b, the downregulation of LINC00961 was detected in 68 cases out of 79 patients (86%). Consistently, LINC00961 was downregulated considerably in two luminal A cell lines namely MCF7, and T47D, compared with normal breast cell line, MCF10A (Fig. 1c). Besides, results obtained from the GEPIA web server across BRCA dataset containing luminal A and B BC samples were consistent with the experimental data of the present study which indicated that LINC00961 was downregulated in luminal A and B BC (Fig. 1d). Moreover, the area under ROC curve (AUC) of LINC00961 was 0.80 in luminal subtype of BC (p-value < 0.001, Fig. 1e). Also, the AUC of this lncRNA was 0.083 in luminal A (p-value < 0.001) and 0.67 (p-value > 0.05) in luminal B BC patients, which needs to be evaluated on more luminal B samples.

### Correlation between LINC00961 expression level and the clinicopathological features

As shown in Table 2, low expression of LINC00961 was significantly correlated with smoking status (p-value = 0.008). Non-smoking patients, that consisted a larger group in the present study, were mostly among the group with high levels of LINC00961. Moreover, according to Table 3, LINC00961 expression level has been associated with age of menarche (p-value = 0.012).

**Table 2:** Correlation of LINC00961 expression with clinicopathological features in BC patients

Characteristics	No. of cases	LINC00961 expression		p-value
		Low (N = 40)	High (N = 39)	
Age at diagnose				
≤ 40	20	11 (28.9%)	9 (24.3%)	0.651
> 40	55	27 (71.1%)	28 (75.7%)	
Tumor size (cm)				
< 2	21	7 (18.9%)	14 (35.9%)	0.248
2–5	41	22 (59.5%)	19 (48.7%)	
> 5	14	8 (21.6%)	6 (15.4%)	
Group				
Luminal A	65	33 (82.5%)	32 (82.1%)	0.958
Luminal B	14	7 (17.5%)	7 (17.9%)	
Grade				
1	10	5 (12.5%)	5 (12.8%)	0.997
2	55	28 (70.0%)	27 (69.2%)	
3	14	7 (17.5%)	7 (17.9%)	
Stage				
I	6	2 (5.3%)	4 (11.4%)	0.466
II	38	22 (57.9%)	16 (45.7%)	
III	29	14 (36.8%)	15 (42.9%)	
Pathology of tumors*				
DCIS	1	1 (4.3%)	0 (0.0%)	0.415
IDC	38	21 (91.3%)	17 (94.4%)	
ILC	1	0 (0.0%)	1 (5.6%)	
Others	1	1 (4.3%)	0 (0.0%)	
Progesterone receptor				
Negative	10	4 (10.0%)	6 (15.4%)	0.472
Positive	69	36 (90.0%)	33 (84.6%)	
Estrogen receptor				
Negative	0	0 (0.0%)	0 (0.0%)	–
Positive	79	40 (100.0%)	39 (100.0%)	
HER2				
Negative	70	35 (87.5%)	35 (89.7%)	0.754
Positive	9	5 (12.5%)	4 (10.3%)	
Smoking^†^				
No	41	18 (81.8%)	23 (95.8%)	0.012*
Yes	5	4 (18.2%)	1 (4.2%)	

^†^Fisher exact test was used for calculating p-value

*p < 0.05

**Table 3:** Correlation of LINC00961 expression with clinicopathological features in BC patients

Characteristics	LINC00961 Expression						p-value
	Low			High			
	N	Mean	SD	N	Mean	SD	
Ki67	25	13.74	5.04	2	13.62	7.63	0.948
P53	14	4.92	4.24	18	9.71	16.00	0.286
BMI	8	28.16	2.55	11	29.10	4.72	0.617
Age of menarche	13	13.07	0.75	17	14.29	1.26	0.004**

**P < 0.01

### Differential expression analysis of LINC00961

Using Gene Expression Atlas software, the baseline expression of LINC00961 in breast tissues was displayed in a heatmap studied across different experiments. The expression level of LINC00961 in normal breast tissues was approximately low to medium with 5 to 19 transcripts per million (TPM) (Additional file 1). Evaluation of the LINC00961 expression among 12 breast cancer categories, including 1910 luminal A and B samples, across Affymetrix Human Genome U133 Plus 2.0 Array platform demonstrated that LINC00961 was downregulated in BC categories compared with normal breast samples (Additional file 2). Also, expression analysis of this lncRNA in 12 luminal BC cell lines showed that LINC00961 was also downregulated in luminal BC cell lines, including MCF-7 and T47D, compared with normal breast cell lines (Additional file 3).

### Co-expression gene network analysis

**Fig. 2:**
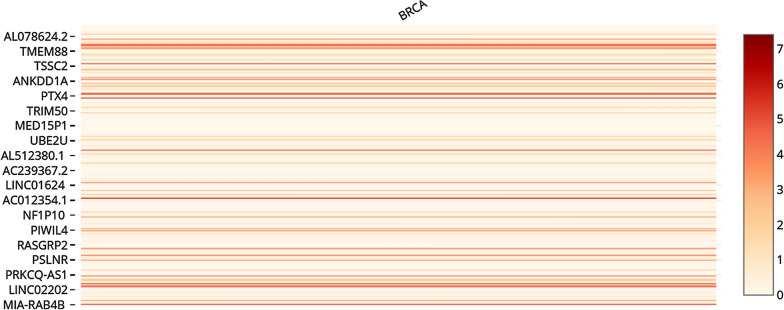
The expression analysis of LINC00961 co-expressed genes across luminal A and B BC. The figure was obtained from the GEPIA web server

According to co-expression analysis of LINC00961, this lncRNA is significantly co-expressed with 358 genes across 420 luminal A and B samples (R ≥ 0.5) (Additional file 4). Expression levels of these co-expressed genes with LINC00961 across BRCA dataset were illustrated in Fig. 2. The results obtained from expression level analysis of the co-expressed genes with LINC00961 were consistent with the data retrieved from dysregulation analysis of LINC00961 in luminal BC. According to Fig. 2, the expression levels of most of the co-expressed genes with LINC00961 were at low levels in BRCA. Detailed expression levels are available at Additional file 5.

### Genetic alterations of LINC00961 across breast cancer

Between 33 cancer types analyzed in the TCGA dataset, LINC00961 wasn’t significantly focally amplified or deleted in breast cancer. Moreover, according to the ICGC data portal, most of the mutations of LINC00961 in BC take place in downstream, upstream and exonic regions, respectively. Based on the data obtained from the ICGC, all LINC00961 mutations were of the substitution type (Additional file 6), and most of them occur in ER^+^ HER2^−^ subtypes including luminal A and B BC.

### Differential analysis

According to expression analysis of LINC00961 in different BC subtypes from TANRIC database, expression of this lncRNA in luminal A, luminal B and HER2 subtypes is lower than the other groups (p-value = 5.31e−45) (Additional file 7).

### Functional annotation analysis

**Fig. 3:**
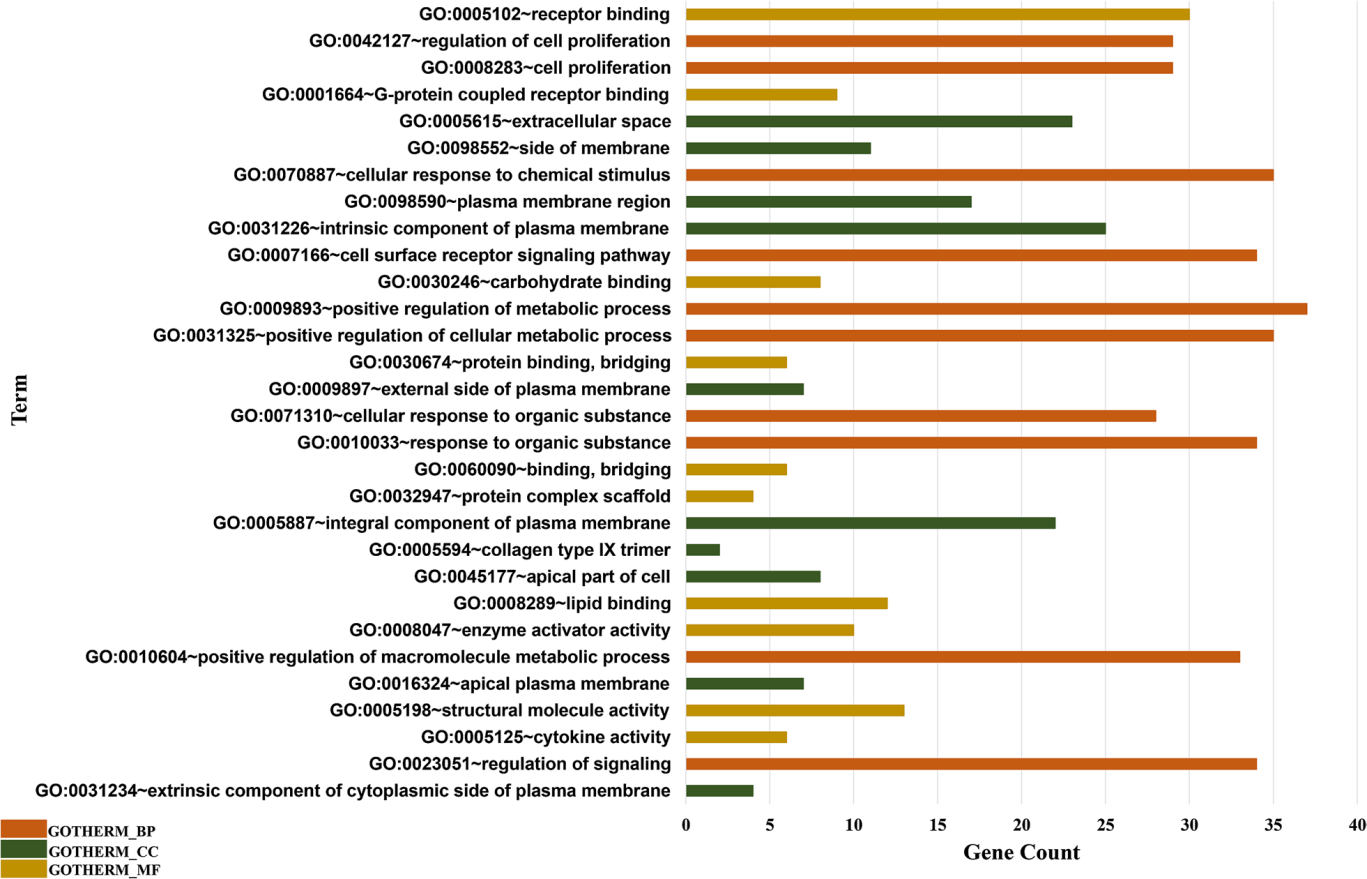
Top 10 gene ontology (GO) terms for categories of biological process (BP), cellular component (CC), and molecular function (MF). Top 10 significant GO terms were selected based on the gene count and the chart was illustrated according to the data obtained from DAVID GO term enrichment analysis. Also, the columns were sorted from top to bottom based on how significant that term is (p‐value < 0.05). GOTERM_BP, GOTERM_CC, and GOTERM_MF terms are representative of biological process, cellular component, and molecular function, respectively

The Gene Ontology (GO) analysis consists of different terms for characterizing the activity and function of various genes. A list of GO terms for biological process (BP), cellular component (CC), and molecular function (MF) were obtained as a result of GO term enrichment analysis performed by DAVID. The significant GO terms (p‐value < 0.05) of these three groups were listed in Additional file 8. The top 10 BP, CC, and MF terms are shown in Fig. 3. According to the GO term enrichment analysis, the genes co-expressed with LINC00961 were particularly involved in various biological processes, such as regulation of cell proliferation, cellular response to chemical stimulus, cell surface receptor signaling pathway, regulation of signaling, and so on. Molecular functions of the co-expressed genes with LINC00961 mostly consist of receptor binding, G-protein coupled receptor binding, carbohydrate and lipid binding, protein complex scaffold, enzyme activator activity, etc. Each of the mentioned activities is executed in a location inside or in the vicinity of a cell. The cellular component terms could help to predict the location of different genes and regarding LINC00961, cellular component terms contained extracellular space, intrinsic component and external side of plasma membrane. The data obtained from summarizing and excluding redundant GO terms by REVIGO suggested similar results to the outcome of DAVID GO enrichment analysis (Additional files 9, 10 and 11).

**Fig. 4:**
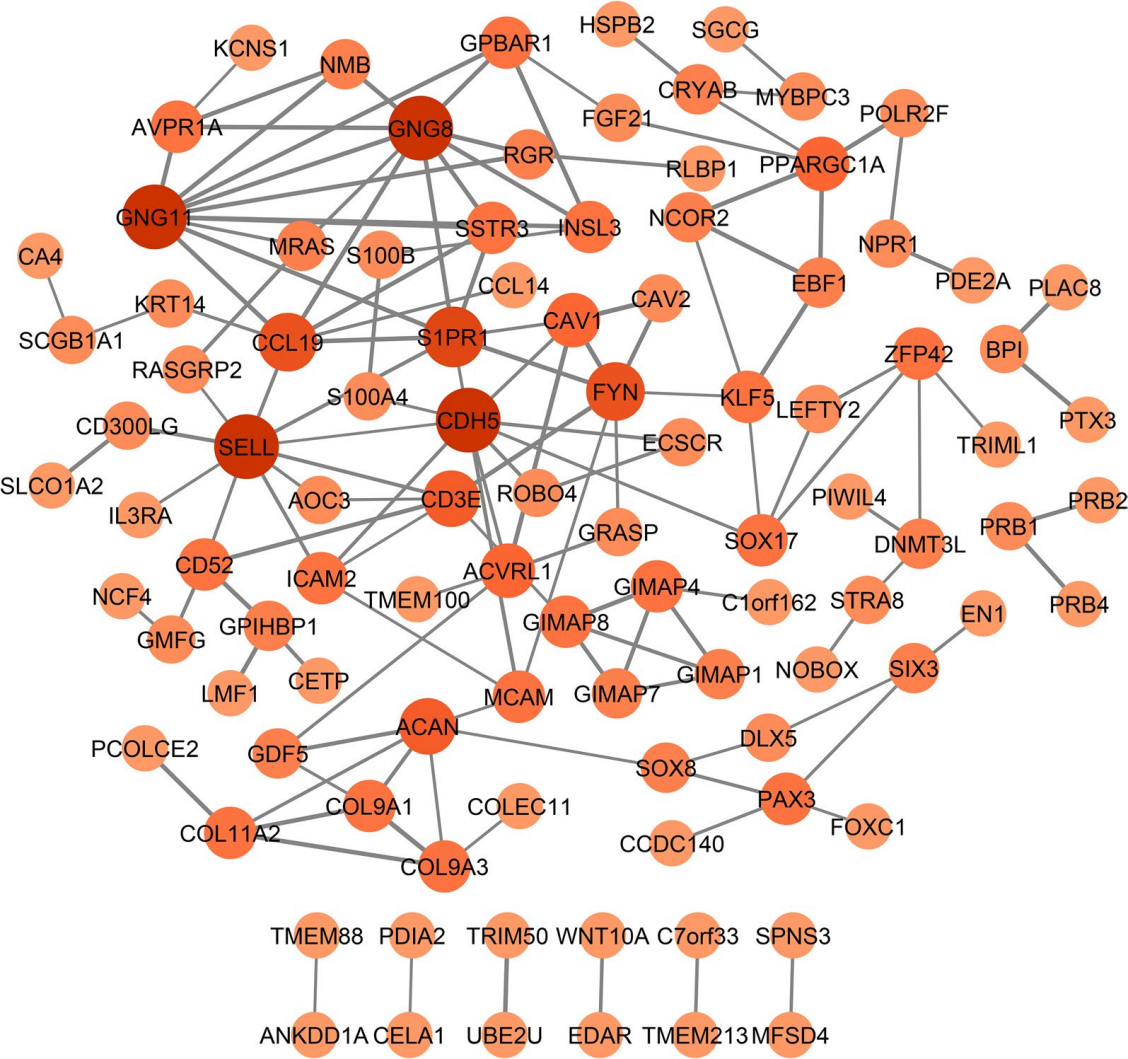
The PPI network of the co-expressed genes with LINC00961, illustrated by Cytoscape. The thicker edge and the stronger node color indicate the higher STRING combined score and the higher degree, respectively. Results were retrieved from the STRING database

Finally, using STRING database, it was found that of the 358 co-expressed genes with LINC00961 across luminal subtype of BC, 100 genes have strong interactions (Interaction score > 0.4) with each other (Fig. 4). According to the CytoHubba, the genes CDH5, GNG11, GNG8, SELL, S1PR1, CCL19, FYN, ACAN, CD3E, ACVRL1, CAV1, and PPARGC1A were adopted as the top 12 genes ranked by degree. According to the GEPIA web server, 7 genes (CDH5, GNG11, SIPR1, FYN, ACVRL1, CAV1, and PPARGC1A) out of the mentioned hub genes of LINC00961 PPI network were significantly downregulated in luminal A and B BC (Additional file 12). Moreover, KEGG, Reactome and WikiPathways analysis by Enrichr showed that the nodes were mainly involved in several cancer-associated pathways, including chemokine, Ras, and PI3K–Akt signaling pathways, GPCR ligand binding, and signal transduction (Table 4).

**Table 4:** The top 10 KEGG, WikiPathway, and Reactome terms enriched by the node genes

Pathway	p-value	Node genes
KEGG		
Chemokine signaling pathway	2.67E−03	CCL14;GNG8;CCL19;RASGRP2;GNG11
Viral myocarditis	3.22E−03	CAV1;FYN;SGCG
Focal adhesion	3.25E−03	CAV2;CAV1;COL9A1;COL9A3;FYN
Proteoglycans in cancer	3.39E−03	WNT10A;MRAS;CAV2;CAV1;HSPB2
Cytokine–cytokine receptor interaction	3.60E−03	EDAR;ACVRL1;CCL14;IL3RA;CCL19;GDF5
Apelin signaling pathway	4.97E−03	MRAS;GNG8;PPARGC1A;GNG11
Ras signaling pathway	6.21E−03	MRAS;GNG8;RASGRP2;GNG11;FGF21
PI3K–Akt signaling pathway	8.73E−03	IL3RA;COL9A1;GNG8;COL9A3;GNG11;FGF21
Protein digestion and absorption	1.04E−02	COL11A2;COL9A1;COL9A3
Morphine addiction	1.02E−02	PDE2A;GNG8;GNG11
WikiPathway		
Preimplantation Embryo	2.03E−04	DNMT3L;SIX3;ZFP42;SOX8
Gastric Cancer Network 2	4.92E−04	PLAC8;COL9A1;COL9A3
Adipogenesis	4.13E−03	NCOR2;KLF5;EBF1;PPARGC1A
Focal Adhesion-PI3K–Akt–mTOR-signaling pathway	4.17E−03	IL3RA;COL11A2;GNG8;PPARGC1A;GNG11;FGF21
Mesodermal Commitment Pathway	6.37E−03	FOXC1;KLF5;SOX17;LEFTY2
Differentiation of white and brown adipocyte	6.89E−02	PLAC8;PPARGC1A
PI3K–Akt Signaling Pathway	7.23E−03	IL3RA;COL9A1;GNG8;COL9A3;GNG11;FGF21
Viral Acute Myocarditis	8.62E−0.3	CAV1;FYN;SGCG
Statin Pathway	9.20E−03	CETP;PDIA2
Chemokine signaling pathway	9.29E−03	GNG8;CCL19;RASGRP2;GNG11
Reactome		
GPCR ligand binding Homo sapiens	1.46E−05	WNT10A;NMB;S1PR1;GNG8;GPBAR1;AVPR1A; CCL19;RGR;INSL3;GNG11;SSTR3
Class A/1 (Rhodopsin-like receptors) Homo sapiens	2.18E−04	NMB;S1PR1;GPBAR1;AVPR1A;CCL19; RGR;INSL3;SSTR3
Collagen biosynthesis and modifying enzymes Homo sapiens	2.79E−04	PCOLCE2;COL11A2;COL9A1;COL9A3
Signal Transduction Homo sapiens	3.73E−04	NCF4;RASGRP2;RLBP1;CDH5;SOX17;S1PR1;GNG8;POLR2F;FYN;CCL19;INSL3;WNT10A;NMB;CAV1;PDE2A;GPBAR1;AVPR1A;S100B;GNG11;SSTR3;NCOR2;IL3RA;COL9A1;COL9A3;RGR
Extracellular matrix organization Homo sapiens	5.50E−04	ACAN;PCOLCE2;COL11A2;COL9A1;ICAM2;COL9A3;GDF5
Transcriptional regulation of white adipocyte differentiation Homo sapiens	6.63E−04	NCOR2;KLF5;EBF1;PPARGC1A
G alpha (s) signaling events Homo sapiens	7.37E−04	PDE2A;GNG8;GPBAR1;INSL3;GNG11
Collagen formation Homo sapiens	8.74E−04	PCOLCE2;COL11A2;COL9A1;COL9A3
GPCR downstream signaling Homo sapiens	1.21E−03	NMB;PDE2A;GPBAR1;AVPR1A;RASGRP2;GNG11;SSTR3;IL3RA;S1PR1;GNG8;CCL19;RGR;INSL3
G alpha (i) signaling events Homo sapiens	1.31E−03	S1PR1;GNG8;CCL19;RGR;GNG11;SSTR3

## Discussion

The luminal subtype of BC accounts for more than 70% of this cancer [4, 8]. LncRNAs have been identified as novel diagnostic and prognostic biomarkers in luminal subgroups of BC. For instance, lncRNA BC200, LOL and DSCAM-AS1 play important roles in resistance to therapy, proliferation, and invasion of luminal BC cells [37–39]. Despite all the research that has been done on dysregulated lncRNAs and their clinical potential as biomarkers, the role of LINC00961 in luminal subtype of BC is largely unknown.

In the present study, LINC00961 was identified as a novel dysregulated lncRNA in luminal BC. LINC00961 was significantly downregulated in luminal A and B BC tissues and luminal A cell lines (T47D, MCF7), compared with their adjacent non-tumoral tissues and non-malignant breast cell line (MCF10A), respectively. Downregulation of LINC00961 was detected in 86% of cases, which is a considerable fraction. The upregulation of this lncRNA in the remaining 14% of the samples may be due to lncRNA's inter-individual expression diversity [40]. Moreover, the result of qRT-PCR assay was consistent with the GEPIA dataset and demonstrated the downregulation of LINC00961 in luminal A and B BC.

LINC00961 expression was negatively correlated with smoking in luminal BC patients. Also, LINC00961 expression correlated with the age of menarche in luminal BC patients. However, due to the influence of other genetic and environmental factors in the age of menarche, more research is needed on this factor.

Further analysis of the expression and potential role of LINC00961 in luminal BC was performed by obtaining a combination of various databases. According to subtype PAM50 classification from TANRIC database, LINC00961 expression level was lower in luminal A, luminal B and HER2 subtypes compared to other subtypes of BC. Also, results obtained from different datasets in GENEVESTIGATOR and GEPIA web server were consistent with the data of our study which indicated downregulation of LINC00961 in luminal BC tissues and cell lines.

According to the data obtained from the TCGA copy number portal, LINC00961 has not been significantly amplified or deleted in invasive breast carcinoma dataset. Also, it was found that all LINC00961 mutations in BC were substitutions occurring more frequently in ER^+^ HER2^−^ patients, including luminal A and B BC patients. It is proved that different substitution mutations could have various effects on the secondary structure of RNAs. On the other hand, RNA secondary structure plays vital roles in RNA regulation, particularly in the miRNA’s interactions and RNA-binding proteins [41]. Also, some evidences suggested that somatic mutations within lncRNAs may contribute to the pathogenesis of various cancers [42]. Therefore, studying the effect of these mutations on the secondary structure of this lncRNA in the future can be very useful in determining the role of this lncRNA in breast cancer.

According to the “guilt-by-association” principle, it is probable that a set of genes which is involved in a same biological pathway are controlled by a common regulatory system and therefore, are co-expressed with each other [43]. In other words, genes involved in similar and/or related biological pathways may be co-expressed across distinct experimental conditions. As a result, Gene Co-expression Network analysis is a very practical tool in predicting function of unknown genes at a genome-wide scale [44]. According to this principle, LINC00961 might be involved in various biological processes in luminal BC, like regulation of cell proliferation, cellular response to chemical stimulus, cell surface receptor signaling pathway, regulation of signaling, and so on. Dysregulation of all of these processes can lead to the development of various cancers. Various signaling pathways, including the WNT signaling pathway, have been shown to be aberrantly activated during breast tumor progression, promoting the metastasis of these cells [45]. Also, it was demonstrated that ncRNAs play essential roles in regulating specific signaling pathways, such as WNT and TGF-β [46]. The molecular function analysis suggested that LINC00961 might be involved in receptor binding, G-protein coupled receptor binding, carbohydrate and lipid binding, protein complex scaffold, enzyme activator activity, etc. Disruption of these processes also can lead to different malignancies. Overwhelming evidence suggests that G protein-coupled receptors (GPCRs) play critical roles in metastasis and invasion of tumor cells. Also, they can control abnormal growth and survival of cancer cells by activating various signaling pathways, including AKT/mTOR and MAPK pathways [47]. Cellular component analysis demonstrated that LINC00961 is mostly located in extracellular space, intrinsic component and external side of plasma membrane. The extracellular matrix (ECM) is an important regulator of BC cells and includes proteins such as fibrillar collagens, fibronectin, laminins and proteoglycans which are induced in breast cancer. A great number of these induced ECM proteins have different roles in metastasis and progression of BC cells [48].

Data obtained from the STRING database illustrated that 100 of the co-expressed genes with LINC00961 had significant interactions with each other. These data confirmed the hypothesis that LINC00961 and its co-expressed genes are involved in the same biological pathways. These genes were shown to be mostly involved in multiple cancer-associated pathways, such as chemokine, Ras and PI3K–Akt signaling pathways, GPCR ligand binding, and signal transduction. Chemokine signaling has been shown to enhance cell migration and metastasis in BC. For example, CCL2/CCR2 chemokine signaling has been overactive in several cancers, including breast cancer, and has been associated with poor prognosis [49]. Also, CCL2 is overexpressed in luminal B subtype of BC and positively regulates the growth of these cells [50]. Moreover, activation of Ras signaling pathway is a key determinant for poor prognosis and dissemination of luminal BC cells in humans [51]. The PI3K–Akt pathway, with frequent PIK3CA mutations in the luminal subtypes of BC, is the most altered pathway in this cancer. It has been suggested that luminal B tumors with hyperactive PI3K signaling are associated with endocrine therapy resistance [52, 53]. As mentioned earlier, GPCR signaling pathways can also play important roles in BC development. These data suggest that LINC00961 may play important roles in luminal BC, by involving in the mentioned pathways. However, further research is needed to determine the involvement and the exact role of LINC00961 in these pathways across luminal BC.

Taken together, to the best of our knowledge, the present study is the first project that proves the dysregulation of LINC00961 in luminal A and B subtype of BC so far. Also, the bioinformatics analysis performed in this study could provide basic information about the potential role of lncRNA LINC00961 in luminal BC. Nonetheless, the current study had some limitations related to functional analysis which should be considered to provide a detailed insight into the mechanism of downregulation of LINC00961 in breast carcinogenesis. Another limitation was the lack of complete overall survival data of BC patients. Investigating whether LINC00961 can be used as a prognostic biomarker for luminal subtypes of BC require data on the overall survival of BC patients in the future follow-up studies.

## Conclusion

In conclusion, according to the q-RT PCR assay and the bioinformatics analyses, LINC00961 was considerably downregulated in luminal A and B BC tissues and cell lines. Also, according to different datasets, LINC00961 might be involved in various cancer-related processes, such as chemokine, Ras and PI3K–Akt signaling pathways, and GPCR ligand binding which still requires more detailed research. Collectively, the findings of the present study could provide a new insight into the possible role of LINC00961 in luminal subtype of breast cancer.

## Supplementary information

**Additional file 1: Figure S1. **The baseline expression of LINC00961 across normal breast tissues, obtained using Gene Expression Atlas software.

**Additional file 2: Figure S2.** LINC00961 expression level across 12 breast cancer categories, including 1910 luminal A and B samples, compared to normal breast tissue, obtained by GENEVESTIGATOR software.

**Additional file 3: Figure S3.** LINC00961 expression level across 13 luminal A and B breast cancer cell lines, compared to 3 normal breast cell lines, obtained by GENEVESTIGATOR software.

**Additional file 4: File S1.** The list of co-expressed genes with LINC00961, across luminal A and B samples.

**Additional file 5: File S2.** The expression level of the co-expressed genes with LINC00961.

**Additional file 6: File S3.** The list of LINC00961 mutations across breast cancer datasets, obtained using the ICGC portal.

**Additional file 7: Figure S4.** Differential LINC00961 expression analysis across different subtypes of breast cancer, obtained using TANRIC database.

**Additional file 8: File S4.** Some of the significant GO terms associated to biological process, molecular function and cellular component of the LINC00961 co-expressed genes, obtained using DAVID database.

**Additional file 9: Figure S5.** The summarization of all GO terms related to biological process. Each node is a GO term and node color indicates the statistical significance of p‐value. Node size suggests how frequent the GO term is among the complete set. The summarization and visualization were done by the REViGO and Cytoscape, respectively.

**Additional file 10: Figure S6.** The summarization of all GO terms related to cellular component. Each node is a GO term and node color indicates the statistical significance of p‐value. Node size suggests how frequent the GO term is among the complete set. The summarization and visualization were done by the REViGO and Cytoscape, respectively.

**Additional file 11: Figure S7.** The summarization of all GO terms related to molecular function. Each node is a GO term and node color indicates the statistical significance of p‐value. Node size suggests how frequent the GO term is among the complete set. The summarization and visualization were done by the REViGO and Cytoscape, respectively.

**Additional file 12: Figure S8.** The expression level of 7 out of 12 hub genes, retrieved by GEPIA web server.

## Data Availability

The datasets supporting the conclusions of this article are available in: [Lnc2Cancer database] at (https://www.bio-bigdata.com/lnc2cancer/). [GDC database] at (https://portal.gdc.cancer.gov/). [Expression Atlas] at (https://www.ebi.ac.uk/gxa/home). [GENEVESTIGATOR software] at (https://www.genevestigator.com). [GEPIA web server] at (https://www.gepia.cancer-pku.cn). [TCGA copy number portal] at (https://www.broadinstitute.org). [International Cancer Genome Consortium] at (https://dcc.icgc.org). [DAVID database] at (https://david.ncifcrf.gov). [REVIGO database] at (https://revigo.irb.hr). [Cytoscape software] at (https://www.cytoscape.org). [STRING database] at (https://string-db.org). Citation of all data is provided in the references list. The datasets supporting the conclusions of this article are also included within the article and its additional files.

## Abbreviations

BC: Breast cancer
LncRNAs: Long non-coding RNAs
DElncRNAs: Differentially expressed LncRNAs
qRT-PCR: Quantitative reverse transcription PCR
PPI: Protein–protein interaction
ER: Estrogen receptor
PR: Progesterone receptor
OSCC: Oral squamous cell carcinoma
HCC: Hepatocellular carcinoma
NSCLC: Non-small cell lung cancer
SPAAR: Small regulatory polypeptide of amino acid response
TCGA: The Cancer Genome Atlas
GDC: Genomic Data Commons
BCRC-BB: Breast Cancer Research Center Biobank
BMI: Body mass index
DEPC: Diethyl pyrocarbonate
β2M: Beta-2-microglobulin
GEPIA: Gene Expression Profiling Interactive Analysis
GTEx: Genotype-Tissue Expression Database
BRCA: Breast invasive carcinoma
SCNA: Somatic copy number alterations
ICGC: International Cancer Genome Consortium
DAVID: Database for Annotation, Visualization and Integrated Discovery
GO: Gene ontology
SD: Standard deviation
ROC: Receiver operating characteristics
AUC: Area under the ROC curve
TPM: Transcripts per million
ECM: Extracellular matrix
FPKM: Fragments per kilobase of exon model per million reads mapped
BP: Biological process
CC: Cellular component
MF: Molecular function
