# Supplementary material for: Evaluation of the potential role of long non-coding RNA LINC00961 in luminal breast cancer: a case–control and systems biology study
Source: Cancer Cell Int. 2020 Oct 2;20:478. doi: 10.1186/s12935-020-01569-1 (PMC7531117; doi:10.1186/s12935-020-01569-1)
Supplement: Supplementary file 1 — Additional file 1: Figure S1. The baseline expression of LINC00961 across normal breast tissues, obtained using Gene Expression Atlas software. [file 12935_2020_1569_MOESM1_ESM.pdf]

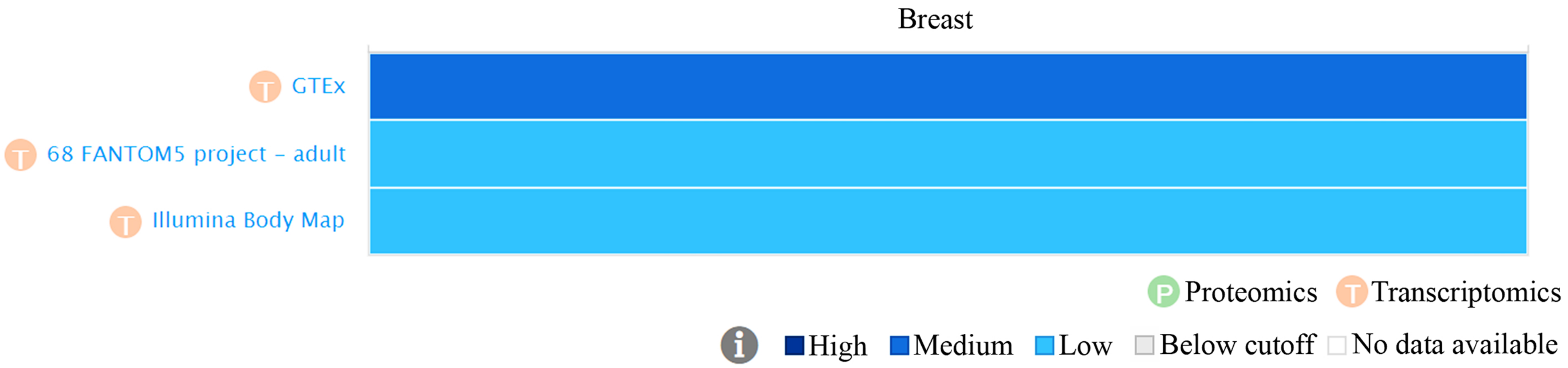
Breast
GTEx
68 FANTOM5 project - adult
Illumina Body Map
Proteomics
Transcriptomics
High
Medium
Low
Below cutoff
No data available
