# Supplementary material for: Evaluation of the potential role of long non-coding RNA LINC00961 in luminal breast cancer: a case–control and systems biology study
Source: Cancer Cell Int. 2020 Oct 2;20:478. doi: 10.1186/s12935-020-01569-1 (PMC7531117; doi:10.1186/s12935-020-01569-1)
Supplement: Supplementary file 2 — Additional file 2: Figure S2. LINC00961 expression level across 12 breast cancer categories, including 1910 luminal A and B samples, compared to normal breast tissue, obtained by GENEVESTIGATOR software. [file 12935_2020_1569_MOESM2_ESM.pdf]

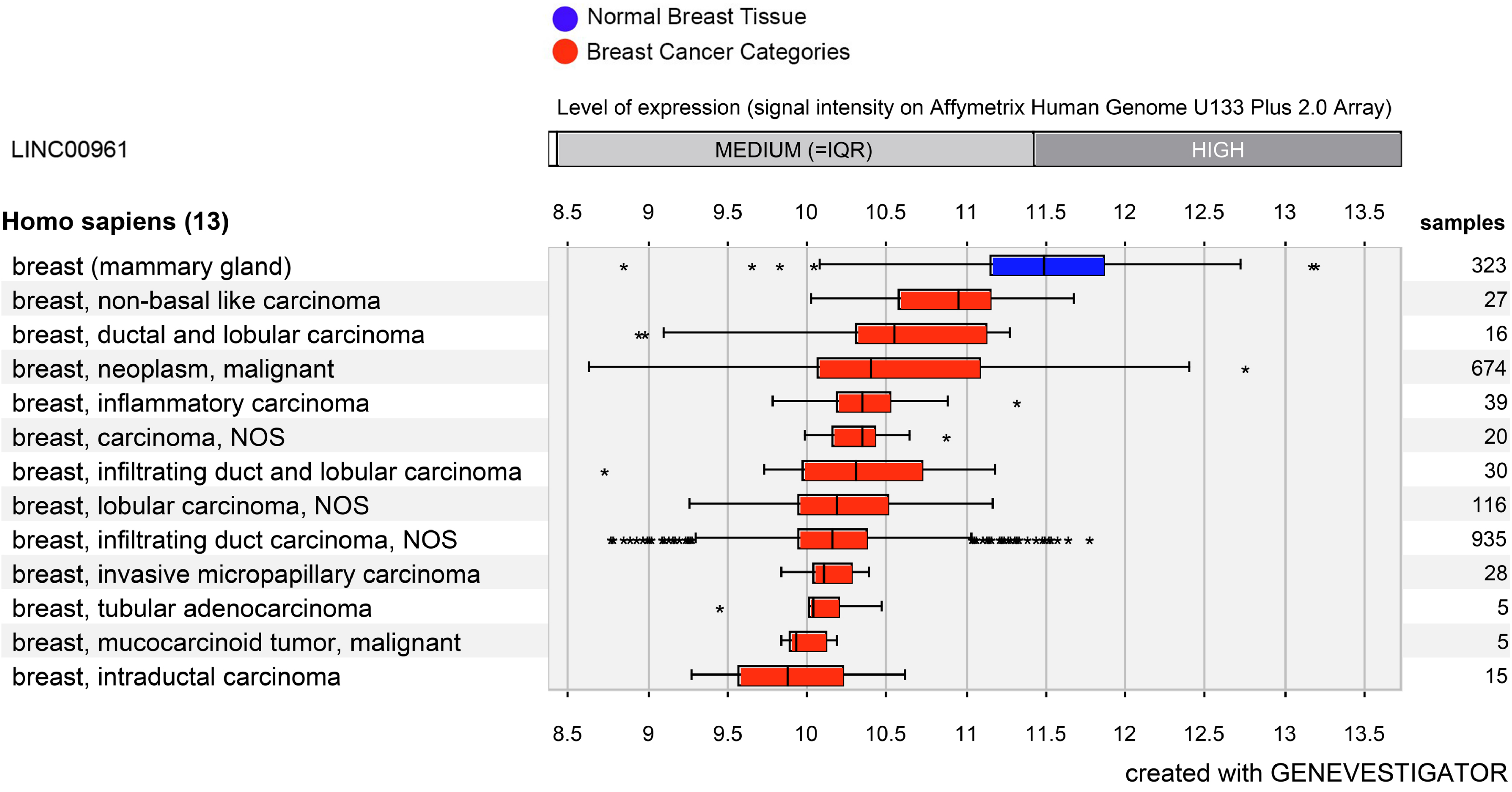
Normal Breast Tissue
Breast Cancer Categories
Level of expression (signal intensity on Affymetrix Human Genome U133 Plus 2.0 Array)
LINC00961
MEDIUM (=IQR)
HIGH
Homo sapiens (13)
8.5 9 9.5 10 10.5 11 11.5 12 12.5 13 13.5
samples
breast (mammary gland) 323
breast, non-basal like carcinoma 27
breast, ductal and lobular carcinoma 16
breast, neoplasm, malignant 674
breast, inflammatory carcinoma 39
breast, carcinoma, NOS 20
breast, infiltrating duct and lobular carcinoma 30
breast, lobular carcinoma, NOS 116
breast, infiltrating duct carcinoma, NOS 935
breast, invasive micropapillary carcinoma 28
breast, tubular adenocarcinoma 5
breast, mucocarcinoid tumor, malignant 5
breast, intraductal carcinoma 15
created with GENEVESTIGATOR
