# Supplementary material for: Evaluation of the potential role of long non-coding RNA LINC00961 in luminal breast cancer: a case–control and systems biology study
Source: Cancer Cell Int. 2020 Oct 2;20:478. doi: 10.1186/s12935-020-01569-1 (PMC7531117; doi:10.1186/s12935-020-01569-1)
Supplement: Supplementary file 3 — Additional file 3: Figure S3. LINC00961 expression level across 13 luminal A and B breast cancer cell lines, compared to 3 normal breast cell lines, obtained by GENEVESTIGATOR software. [file 12935_2020_1569_MOESM3_ESM.pdf]

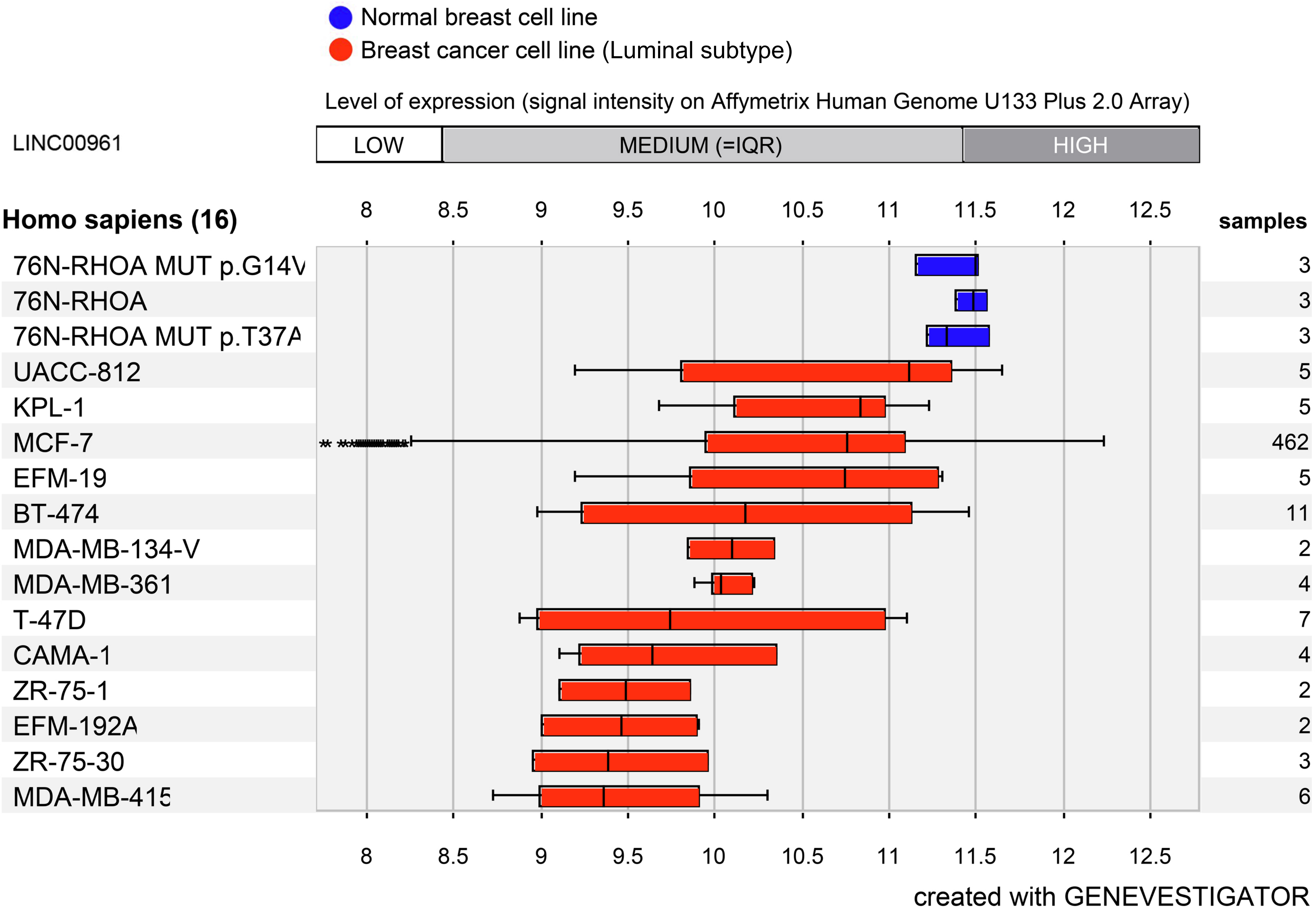

Normal breast cell line
Breast cancer cell line (Luminal subtype)
Level of expression (signal intensity on Affymetrix Human Genome U133 Plus 2.0 Array)
LINC00961
LOW
MEDIUM (=IQR)
HIGH
Homo sapiens (16)
8
8.5
9
9.5
10
10.5
11
11.5
12
12.5
samples
76N-RHOA MUT p.G14V 3
76N-RHOA 3
76N-RHOA MUT p.T37A 3
UACC-812 5
KPL-1 5
MCF-7 462
EFM-19 5
BT-474 11
MDA-MB-134-V 2
MDA-MB-361 4
T-47D 7
CAMA-1 4
ZR-75-1 2
EFM-192A 2
ZR-75-30 3
MDA-MB-415 6
created with GENEVESTIGATOR
