# Supplementary figures and images for: Evaluation of the potential role of long non-coding RNA LINC00961 in luminal breast cancer: a case–control and systems biology study
Source: Cancer Cell Int. 2020 Oct 2;20:478. doi: 10.1186/s12935-020-01569-1 (PMC7531117; doi:10.1186/s12935-020-01569-1)

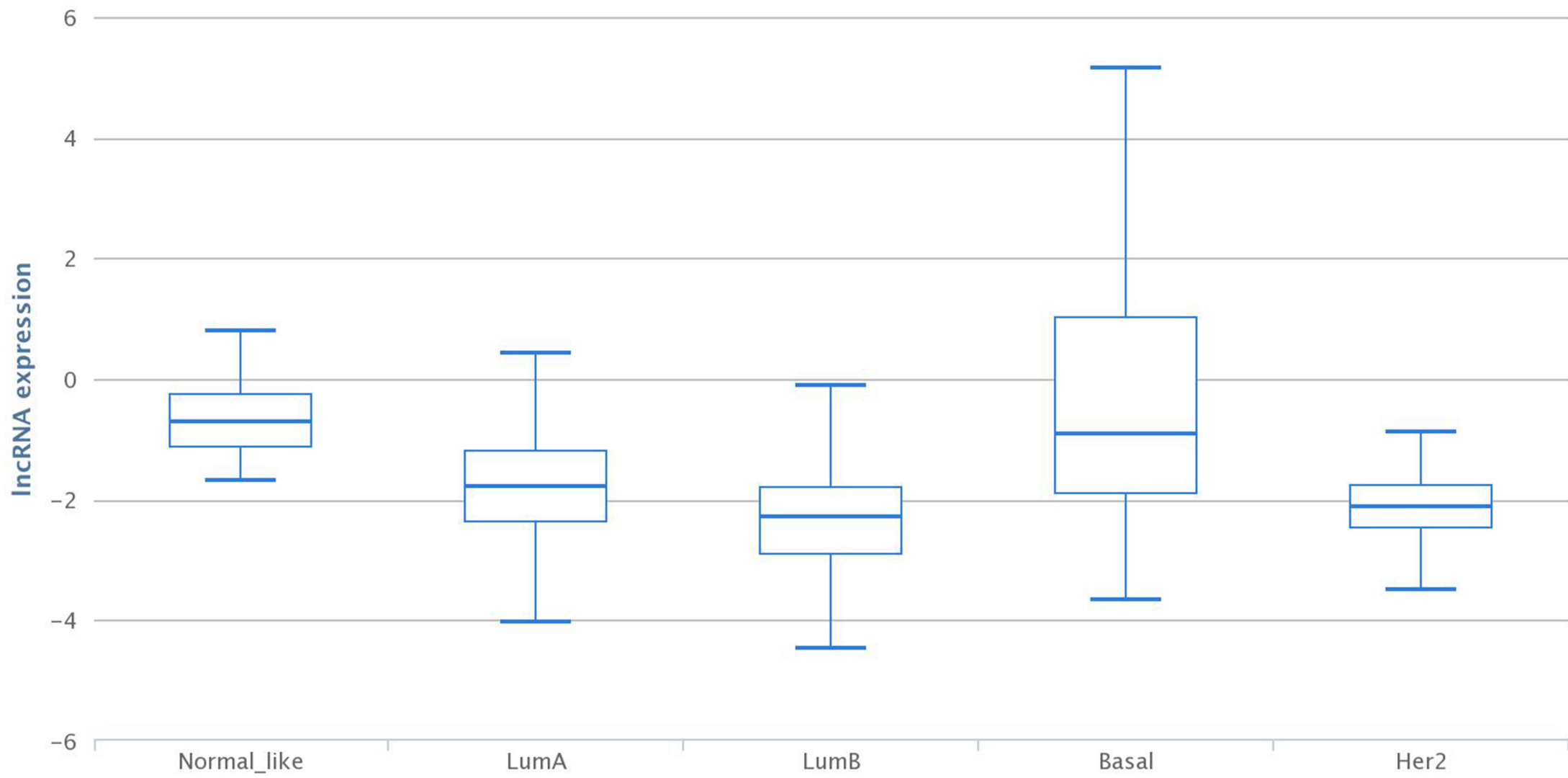

Supplement: Supplementary file 7 — Additional file 7: Figure S4. Differential LINC00961 expression analysis across different subtypes of breast cancer, obtained using TANRIC database. [file 12935_2020_1569_MOESM7_ESM.pdf]

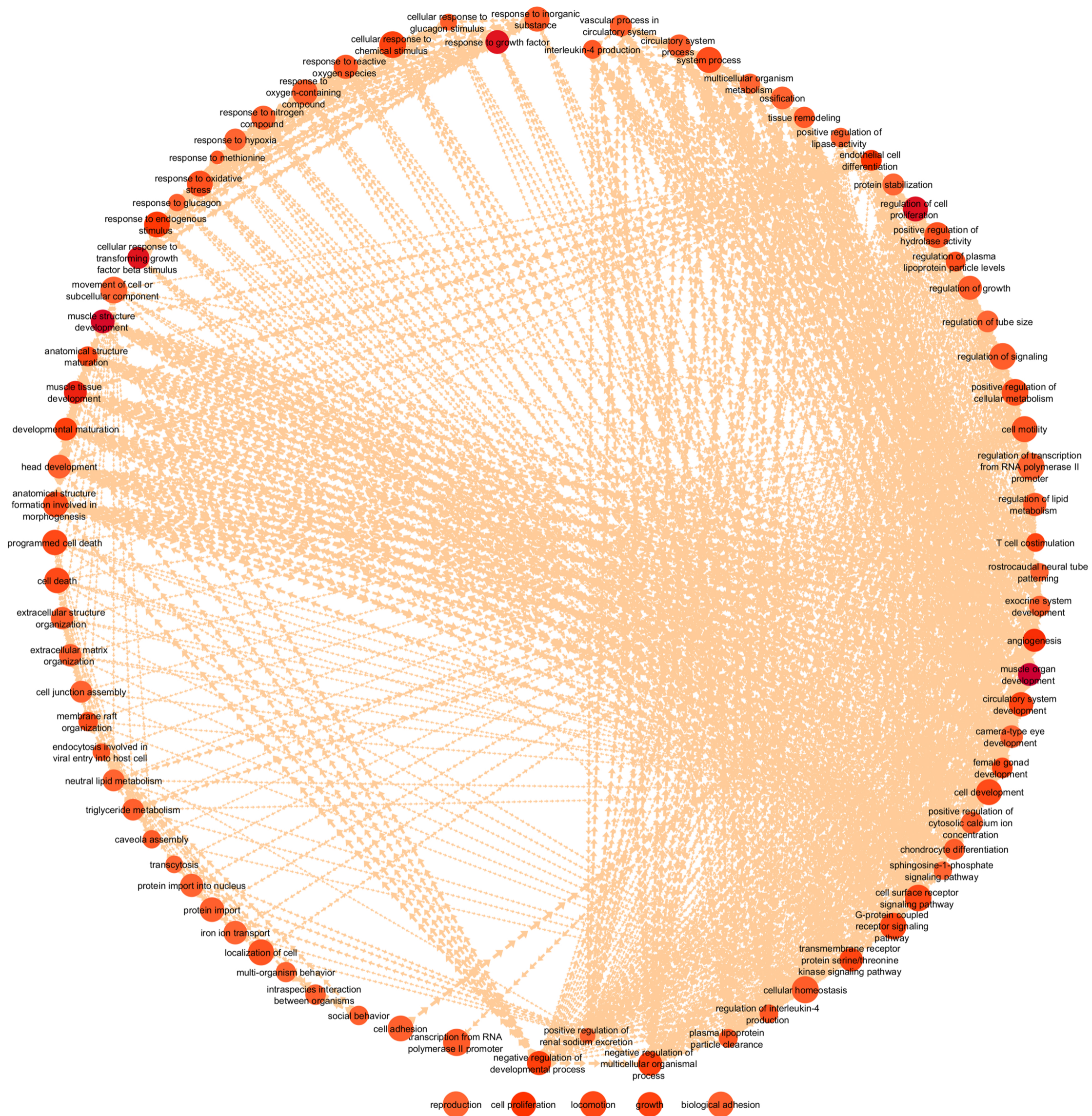

BP-terms of LINC00961 co-expressed genes

Supplement: Supplementary file 9 — Additional file 9: Figure S5. The summarization of all GO terms related to biological process. Each node is a GO term and node color indicates the statistical significance of p‐value. Node size suggests how frequent the GO term is among the complete set. The summarization and visualization were done by the REViGO and Cytoscape, respectively. [file 12935_2020_1569_MOESM9_ESM.pdf]

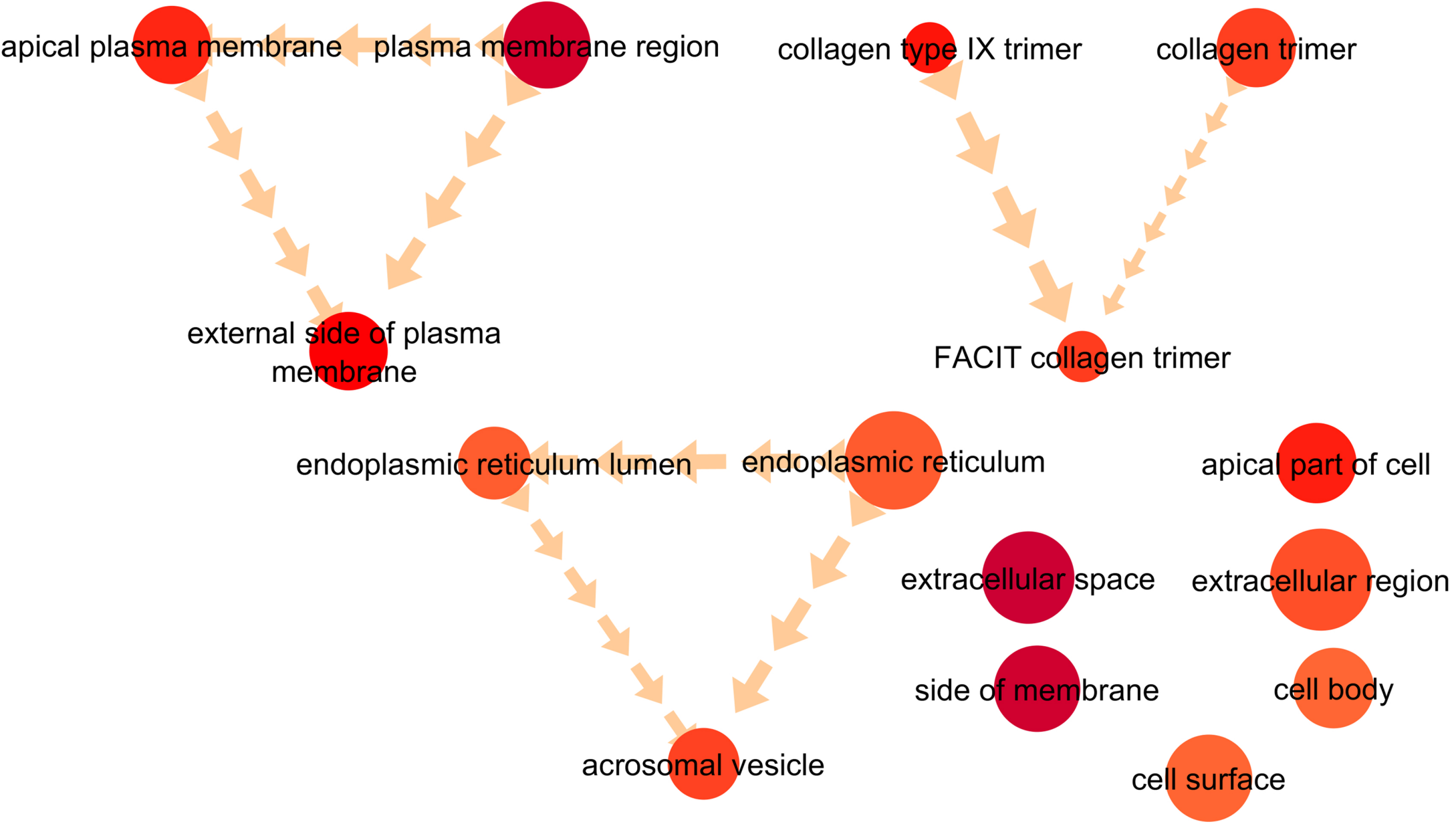

CC-terms of LINC00961 co-expressed genes

Supplement: Supplementary file 10 — Additional file 10: Figure S6. The summarization of all GO terms related to cellular component. Each node is a GO term and node color indicates the statistical significance of p‐value. Node size suggests how frequent the GO term is among the complete set. The summarization and visualization were done by the REViGO and Cytoscape, respectively. [file 12935_2020_1569_MOESM10_ESM.pdf]

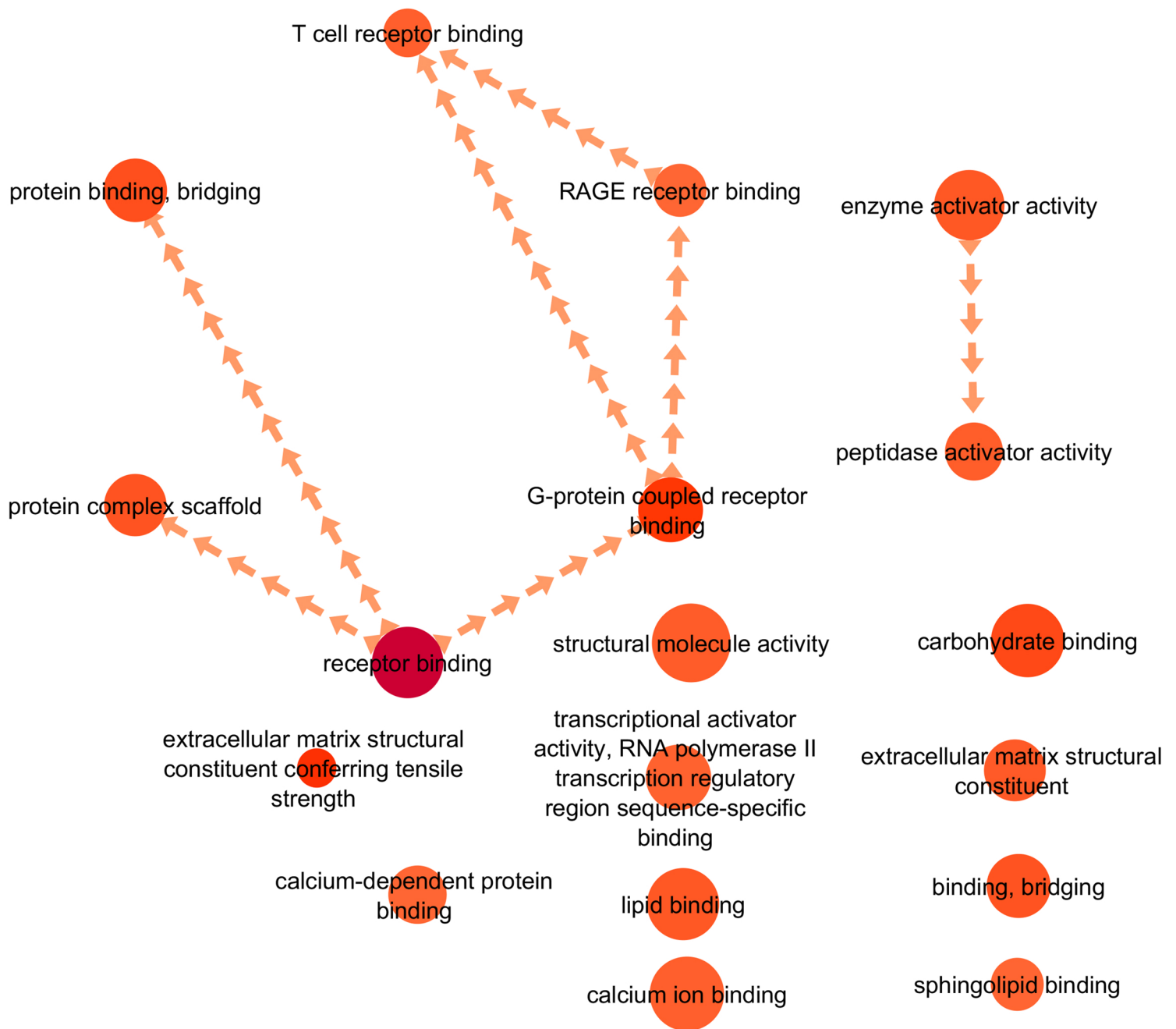

MF-terms of LINC00961 co-expressed genes

Supplement: Supplementary file 11 — Additional file 11: Figure S7. The summarization of all GO terms related to molecular function. Each node is a GO term and node color indicates the statistical significance of p‐value. Node size suggests how frequent the GO term is among the complete set. The summarization and visualization were done by the REViGO and Cytoscape, respectively. [file 12935_2020_1569_MOESM11_ESM.pdf]

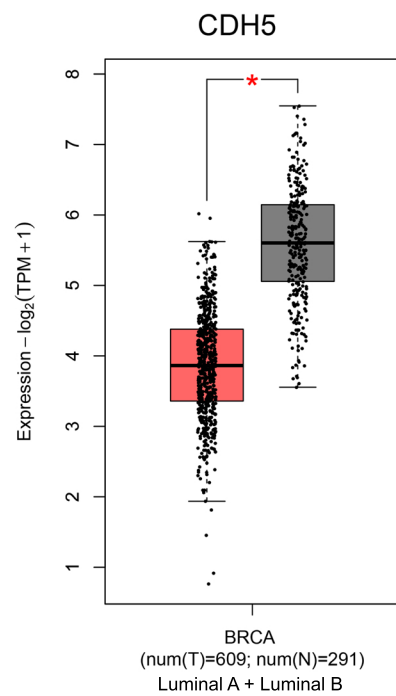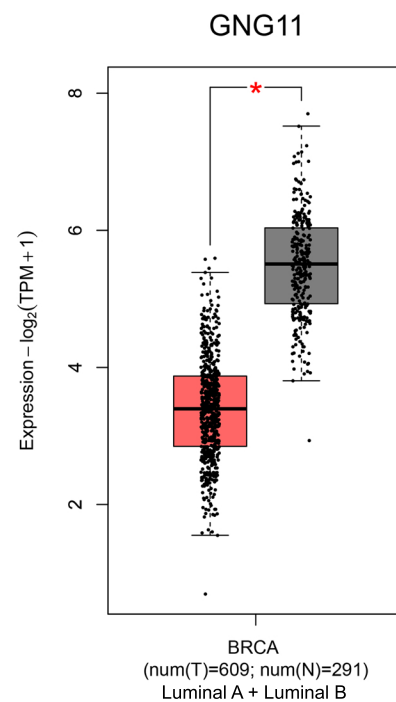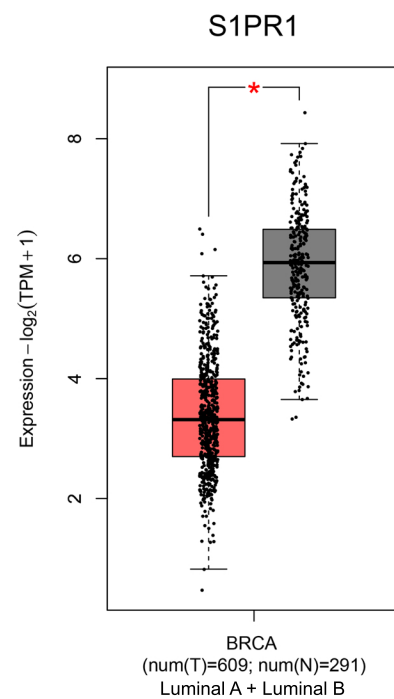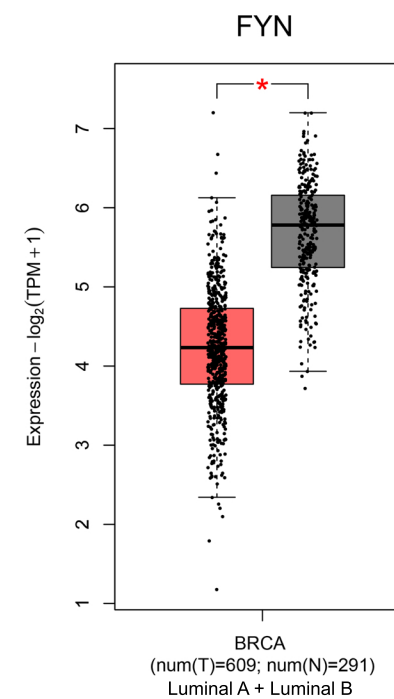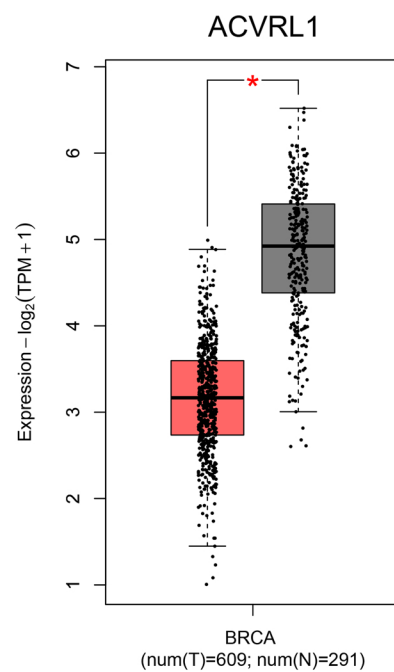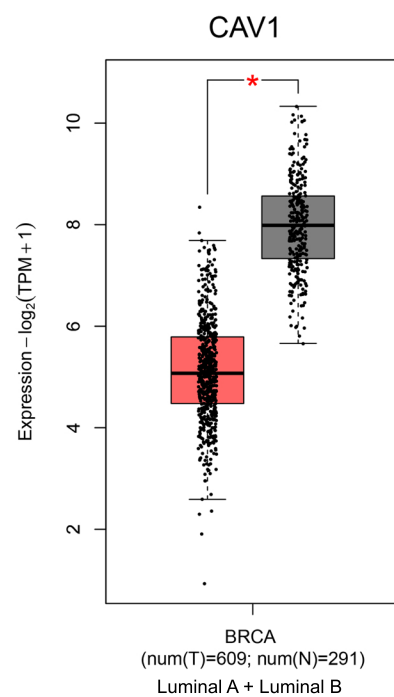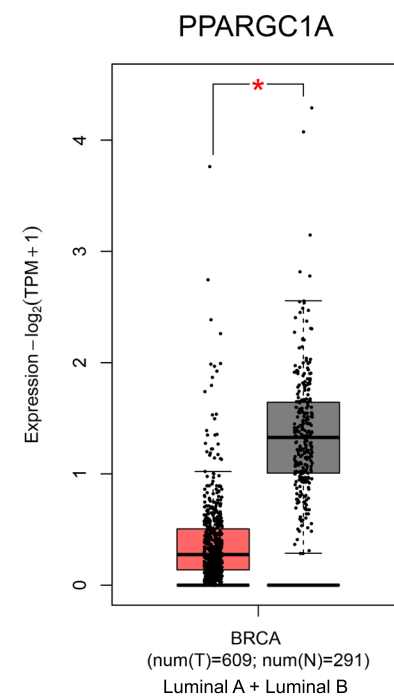

Supplement: Supplementary file 12 — Additional file 12: Figure S8. The expression level of 7 out of 12 hub genes, retrieved by GEPIA web server. [file 12935_2020_1569_MOESM12_ESM.pdf]
